# Chronic Rhinosinusitis in Cystic Fibrosis: Diagnosis and Medical Management

**DOI:** 10.3390/medsci7020032

**Published:** 2019-02-22

**Authors:** Chetan Safi, Zhong Zheng, Emily Dimango, Claire Keating, David A. Gudis

**Affiliations:** 1 Department of Otolaryngology—Head and Neck Surgery, Columbia University Irving Medical Center; New York, NY 10032, USA; chetansafi@gmail.com (C.S.); zz2618@cumc.columbia.edu (Z.Z.); 2 Department of Pulmonary, Allergy, and Critical Care Medicine, Columbia University Irving Medical Center; New York, NY 10032, USA; ead3@cumc.columbia.edu (E.D.); ck2132@cumc.columbia.edu (C.K.)

**Keywords:** rhinosinusitis, cystic fibrosis, diagnosis, medical management

## Abstract

Chronic rhinosinusitis (CRS) is nearly ubiquitous in patients with cystic fibrosis (CF). CF CRS is a challenging entity to define, diagnose, and treat, as patients often have severe refractory sinus disease in addition to complex medical comorbidities. The purpose of this article is to review the literature on the medical management of CF CRS and determine how to best identify, diagnose, and manage CF CRS. Ultimately, the treatment of these patients requires a multi-disciplinary approach involving the pulmonologist and otolaryngologist.

## 1. Introduction

Cystic fibrosis (CF) is an autosomal recessive genetic disorder characterized by poor chloride ion (Cl^-^) transport across cell membranes due to mutations in the cystic fibrosis transmembrane conductance (*CFTR*) gene. It is a systemic disease that can affect the sinopulmonary, gastrointestinal, and genitourinary systems [1]. Reduced chloride transport leads to reduced water crossing the epithelium into mucosal secretions, resulting in thick, inspissated mucus. The pulmonary morbidity of CF is due to this thickened mucus that results in poor mucociliary clearance, secondary bacterial colonization, and recurrent infectious exacerbations that ultimately decrease overall lung function [2]. This continuous process of mucosal inflammation and infection can also affect the paranasal sinuses, leading to chronic rhinosinusitis [3]. Moreover, in considering a unified airway model, some investigators have demonstrated that in CF patients, the paranasal sinuses can serve as a reservoir for virulent bacteria that can then lead to pulmonary exacerbations [4,5,6]. Thus, collaboration with a CF pulmonologist is essential to diagnose and treat chronic rhinosinusitis in these complex patients. In this review, our aim is to identify the best strategies to diagnose cystic fibrosis chronic rhinosinusitis (CF CRS) as well as review the medical therapies available to treat these patients. The indications and benefits of surgical therapy are outside the scope of this review.

## 2. Diagnosis

One challenge in treating CF CRS is defining the disease and identifying patients. The International Consensus Statement on Allergy and Rhinology states that CRS is defined by persistent sinus inflammation for over 12 weeks with the presence of subjective symptoms and objective measures [7]. Subjective symptoms include nasal obstruction, purulent rhinorrhea, facial pain or pressure, and hyposmia or anosmia. Objective findings include purulent sinonasal discharge, mucosal edema, or nasal polyps on nasal endoscopy with evidence of mucosal inflammation on cross-sectional imaging [7]. However, unlike in patients without CF, there is often not a correlation between symptoms and objective measures of sinonasal disease in CF patients. While 60–80% of CF patients have radiographic evidence of CRS, fewer than 20% of patients will spontaneously report symptoms [3,8]. Nevertheless, physicians today still use quality of life metrics, nasal endoscopy, and cross-sectional imaging to assess patients for CF CRS.

## 3. Quality of Life Metrics

CRS symptoms of nasal obstruction, purulent rhinorrhea, facial pain, and hyposmia, can have a dramatic impact on some patients’ quality of life (QOL). Hopkins et al. showed that the Sinonasal Outcome Test-22 (SNOT-22) is a validated instrument that includes rhinologic, extra-nasal rhinologic, aural/facial, psychologic dysfunction, and sleep dysfunction domains that can be used to determine the effect of CRS on a patient’s QOL as well as assess the outcome of surgical therapy [9]. Habib et al. compared CF patients with and without CRS and found that a SNOT-22 score greater than 21 was indicative of concomitant CRS in CF patients [10]. Moreover, the study found that SNOT-22, as a single variable predictor, did not differ from a multivariable regression model, including several sociodemographic and clinical variables, in determining the presence or absence of CRS [10]. Another study found that the respiratory component of the Cystic Fibrosis Questionnaire—Revised for adults and adolescents above age 14, a CF-specific QOL metric, was statistically lower in patients with CRS compared to patients without CRS. This indicates worsened perceived respiratory health in patients with chronic sinus disease [11].

Another QOL instrument studied in the pediatric CF population is the Sinus and Nasal Quality of Life Survey (SN-5). The SN-5 has been validated as a reliable health-related QOL survey in children ages two to fourteen with sinonasal symptoms [12]. It asks parents about their child’s sinus infections, nasal obstruction, allergy symptoms, emotional distress, and activity limitations [12]. Wentzell et al. found that SN-5 scores significantly correlated with recent episodes of sinusitis, antibiotic prescriptions for sinusitis, and number of days missed from school, concluding that this instrument is a reliable method for monitoring sinonasal symptoms in children with CF [13]. Xie et al. found that all 5 domains of the SN-5 correlated with a change in overall QOL scores for children aged zero to four; however, for children aged five to twelve and thirteen to eighteen only two or less subdomains of the SN-5 correlated with overall QOL. They suggested that an overall better instrument be developed to evaluate older children and adolescent CF patients with CRS [14].

## 4. Nasal Endoscopy/Computed Tomography

Nasal endoscopy is another tool commonly used by otolaryngologists in diagnosing paranasal sinus pathology. For CF patients with sinonasal disease, nasal endoscopy can reveal thick nasal drainage, mucosal edema, and nasal polyposis [4,8,15]. However, these findings can be non-specific and do not always correlate with symptoms [15]. Casserly et al. demonstrated that CF patients with and without subjective symptoms of sinus disease had similar Lund–Kennedy nasal endoscopy (LKNE) scores which were overall worse than the general population without CRS. These findings indicate that the inflammatory component of CF can produce a clinically detectable difference in sinus and nasal mucosal health on nasal endoscopy. However, the challenge with CF patients is reconciling this objective data with subjective symptomatology when considering medical or surgical intervention. 

Computed tomography without contrast is indispensable in evaluating patients for CF CRS. Common findings include sphenoid or frontal hypoplasia/aplasia, medial bulging of the lateral nasal wall, demineralization of the uncinate process, sphenoethmoidal recess inflammation, sinus opacification, osteitis and neogenesis, and sclerosis of paranasal sinus bone [16,17,18]. However, as with nasal endoscopy, there is evidence that CT findings do not always correlate with symptoms. Kang et al. found that while 84.7% of their CF cohort had radiologic evidence of CRS, there was no statistically significant difference in Lund-MacKay scores amongst patients stratified based on the severity of their sinonasal symptoms on SNOT-22 [16]. They question whether this dichotomy between imaging and symptoms is a sign of true lack of pathologic inflammation or simply an underreporting of symptoms in the presence of real sinus disease [16]. Furthermore, Rasmussen et al. states that cross-sectional imaging with CT should not be the single determinant when considering ESS as there is no correlation between imaging findings and detection of pus or pathogenic bacteria [19]. Purulence and pathogenic bacteria were found in patients without sinus opacification on CT and sterile cultures were also found in patients with sinus opacification [19]. Due to these imaging uncertainties, some have developed a novel CT scoring system which combines characteristics such as lateral nasal wall bulging and sinus opacification with uncinate demineralization and mucocele presence to quantify severity of sinus disease in CF patients [20]. With respect to children with CF, authors have shown that most patients between 0 and 17 years old will have more severe sinus opacification than their non-CF counterparts [17]. Thus, to limit excessive radiation, CT scans should only be used for peri-operative evaluation and for assessment of sinusitis-related complications [21].

The relationship between CF patients’ genotype and resultant severity of sinus disease has also been assessed. Abuzeid et al. found that patients with high risk CF genotypes, such as class I–III mutations which lead to markedly diminished presence or activity of the cystic fibrosis transmembrane regulator chloride channel, have no statistically significant difference in their SNOT-22, Lund–Mackay, and LKNE scores when compared to low-risk CF genotypes, such as class IV–V mutations, after controlling for confounding variables [22]. The class of mutations are determined based on the mechanism of how they disrupt the *CFTR* gene with categories ranging from defective synthesis of *CFTR* in class I mutations to reduced synthesis or stability of active *CFTR* in class V mutations [22]. On the other hand, Berkhout et al. evaluated CT scans in CF patients and found that those with class I–III mutations had a statistically higher Lund–Mackay score per component of sinonasal system when compared to patients with class IV–V mutations [8]. They noted significantly smaller frontal and sphenoid sinuses, more overall paranasal sinus opacification, and more osteitis/neoosteogenesis of the maxillary sinus wall in the high risk genotype group [8].

Based on the above findings, both nasal endoscopy and computed tomography should be used concurrently to identify objective characteristics of sinonasal disease in CF patients presenting with sinonasal symptoms. The symptomatic patient with evidence of nasal polyposis, sinonasal mucopurulence, and mucosal edema on nasal endoscopy as well sclerotic bone and opacified paranasal sinuses on CT should possibly be treated for CRS.

## 5. Treatment

In treating CF-related sinonasal disease, a number of systemic and topical medical therapies have been identified, with varied data supporting their efficacy as seen in Table 1. Dornase-alfa is a mucolytic agent that functions by cleaving extracellular DNA in the airways and improving mucus viscosity while improving lung function and reducing pulmonary exacerbations [23]. In a randomized controlled trial, CF patients underwent nasal nebulized treatment with dornase-alfa or placebo for 1 year after endoscopic sinus surgery [24]. At 48 weeks after surgery, patients receiving dornase alfa had a statistically significant improvement in their sinonasal symptoms, LKNE score, Lund–Mackay score, and forced expiratory volume in 1 s (FEV1) compared with those receiving placebo [24]. When compared to isotonic nasal saline, Mainz et al. found that CF patients treated with nebulized intranasal dornase-alfa had statistically significant improvement in SNOT-22 scores as well as in forced expiratory flow 75–25% [25].

Nasal saline irrigations are also commonly used to treat chronic sinusitis. Mainz et al. performed a randomized controlled trial in which CF patients used both 28 days of isotonic saline irrigations and 28 days of hypertonic (6.0%) saline irrigations with a washout period in-between [26]. In total, 4 mL of saline was administered daily using a nebulizer and was aerosolized into the nasal cavities. Both therapies led to similarly small improvements in SNOT-22 scores, though it is important to consider that hypertonic saline can lead to more irritated mucosa [3,26]. Others have postulated that saline irrigations themselves can be a poor method to clear sinuses of mucus and crusting or to serve as a delivery method for medications such as antibiotics and steroids because of poor sinus penetration. Aanaes et al. found that even after endoscopic sinus surgery, no saline irrigation reached the frontal and sphenoid sinuses as measured by single-photon emission computed tomography, with less than 50% of maxillary sinuses showing an improvement in post-operative sinus fluid volume after irrigation [27].

Topical corticosteroids are another option available in treating CF sinonasal disease. Hadfield et al. performed a randomized controlled trial in which CF patients with nasal polyps were given 6 weeks of either twice daily topical betamethasone or placebo [28]. After six weeks, there was a statistically significant reduction in nasal polyposis but no change in symptom score [28]. In a retrospective series, Donaldson et al. found that 62.5% of CF patients with nasal polyposis had resolution of polyps or smaller polyps after twice daily nasal inhalations of beclomethasone (100 mg) [29]. In the same study, he found that 78.6% of CF patients without nasal polyposis had improvement in nasal obstructive symptoms with the same regimen [29]. Additionally, other authors have demonstrated the efficacy of beclomethasone with respect to nasal polyp size and improved sinus symptomatology [30,31]. However, there is no conclusive data on the use of oral steroids in the CF population when used to manage sinus disease [3]. In terms of safety profile, topical inhaled corticosteroids such as budesonide can be used without causing dysfunction to the hypothalamic-pituitary axis while use of oral corticosteroids should be guarded due to a high incidence of diabetes secondary to pancreatic insufficiency and metabolic disorders in the CF population [3,32,33].

As chronic infection in the upper and lower airways with *Pseudomonas aeruginosa* and *Staphylococcus aureus* are common in CF patients, topical antibiotics are yet another therapy for treating CF-related sinus disease [34]. One study found that after endoscopic sinus surgery, a postoperative regimen of two weeks of broad spectrum intravenous antibiotics, six months of colistin irrigations, and at least 6 months of topical nasal steroids resulted in a negative sinus culture for at least 6 months in about 50 percent of patients [35]. However, it is difficult to determine what degree of reduced colonization can be attributed to surgical versus medical therapy alone. Additionally, in a randomized controlled trial, Mainz et al. found that topical aerosolized tobramycin resulted in a decrease in burden of paranasal sinus *P. aeruginosa* growth as well as a statistically significant improvement in SNOT-20 scores when compared to a placebo [36]. Yet another study found that the re-operation rate for CF patients who underwent serial sinus lavage of antibiotics such as tobramycin after endoscopic sinus surgery versus patients who underwent conventional surgery alone was 10% vs. 47% 1 year postoperatively and 22% vs. 72% 2 years postoperatively, respectively [37]. This evidence suggests that topical antibiotics can be useful in the treatment of CF CRS.

Ivacaftor is a recently developed novel therapy which works as a *CFTR* modulator that acts by restoring *CFTR* ion transport function at the cellular level in patients with specific *CFTR* mutations known as gating mutations [38]. In CF patients with gating mutations, ivacaftor substantially improved baseline lung function, lowered sweat chloride levels to below disease levels, and has slowed deterioration in lung function by nearly fifty percent per year [39,40]. With regards to sinus disease, Chang et al. describes a patient with medically and surgically recalcitrant CF-related sinus disease who developed newly cleared maxillary and frontal sinuses, improved symptomatology, and improved FEV1 after 10 months of ivacaftor therapy [38]. Additionally, in vitro studies also showed evidence of increased ion transport as well as improved viscosity of secretions with ivacaftor [38]. Furthermore, McCormick et al. demonstrated that ivacaftor therapy improved patients’ rhinologic, sleep, and psychological domain scores on the SNOT-20 quality of life metric 1 and 3 months after starting therapy, though it should be mentioned that 75% of patients started with a score of <1 on SNOT-20 [41]. There has since been approval of two additional *CFTR* modulator therapies approved for an ever expanding list of *CFTR* genotypes. Though a surplus of data on the modulators’ effect on CF CRS is still lacking, it is possible that these groundbreaking drugs, with their ability to restore chloride transport to improve mucociliary clearance in CF, will modify the course and attenuate the severity of disease of the upper airway as it does in the lower airways.

Ibuprofen is yet another medical therapy that some have trialed for the treatment of CF-related sinonasal disease. One study showed that 12 patients with CF and nasal polyposis had resolution of their nasal polyps at some point during high-dose ibuprofen therapy [42]. However, more than half of patients who stopped ibuprofen had a recurrence of nasal polyps, likely indicating only a temporary benefit [40]. While multiple studies have shown a pulmonary benefit in the use of ibuprofen for CF patients, studies evaluating its use in concordant CRS are limited to this one study that at best indicates a partial temporary response to nasal polyposis [43,44].

### Our Experience

At our institution, we use a variety of methods to diagnose and treat patients with CF CRS. Our main diagnostic strategies include history, physical exam, nasal endoscopy, and CT of the paranasal sinuses to evaluate for mucosal inflammation and presence of sinus hypoplasia/and osteitic bone. Together, our otolaryngologists and pulmonologists additionally discuss recent pulmonary function and frequency of pulmonary infections to help determine which patients may require treatment. Our therapeutic strategy includes a combination of surgical and medical therapy; the details of surgical therapy are outside the scope of this review. The medical management includes a combination of saline irrigation compounded with a corticosteroid and antibiotics, delivered with a sinus rinse bottle, which is readily available at local pharmacies. Our experience is consistent with the benefits demonstrated in the literature regarding similar strategies. Additionally, many patients referred to our office are concurrently using systemic therapies such as a *CFTR* modulator.

## 6. Conclusions

Cystic fibrosis is a multisystem disease with a high-level of pulmonary morbidity and mortality that also has a considerable impact on the paranasal sinuses. CRS negatively affects quality of life in individuals with CF. Defining CF-related sinonasal disease and determining the indications for medical and surgical intervention remain a challenge. For primary medical therapy to treat CF-related sinus disease, a range of options are available, with the most data supporting the use of dornase alfa, topical corticosteroids, and topical antibiotics. Further prospective trials are needed to determine the optimal medical management for these patients.

## Figures and Tables

**Table 1:** Summary of medical management used to treat cystic fibrosis rhinosinusitis.

Reference	Therapeutic	Duration	Outcome
Cimmino et al. [24]	Dornase-alpha	48 weeks	Improved sinonasal symptoms, LKNE score, LMS, and FEV1
Mainz et al. [25]	Dornase-alpha	4 weeks	Improved SNOT-22 and forced expiratory flow 75%–25%
Mainz et al. [26]	Isotonic vs. hypertonic saline	4 weeks	Both led to similar small improvements in SNOT-22
Hadfield et al. [28]	Beclomethasone	6 weeks	Reduction in polyposis but no change in symptom score
Donaldson et al. [29]	Beclomethasone	Not described	Improvement in nasal polyposis and nasal obstruction
Aanaes et al. [35]	Colistin irrigations	6 months	Negative sinus cultures for at least 6 months in at least 50% of patients
Mainz et al. [36]	Nebulized tobramycin	4 weeks–8 weeks	Decreased burden of *P. aeruginosa* and improvement in SNOT-22
Moss et al. [37]	Tobramycin lavage	7–10 days (with repetitive treatments if necessary)	Improved reoperation rate for endoscopic sinus surgery
Chang et al. [38]	Ivacaftor	10 months	Improved symptoms and FEV1
Lindstrom et al. [40]	Ibuprofen	Unknown	Temporary resolution of nasal polyps with >50% of patients experiencing recurrence

Abbreviations, LKNE: Lund–Kennedy Nasal Endoscopy, LMS: Lund–Mackay score, FEV1: forced expiratory volume in 1 s, SNOT-22: Sinonasal Outcome Test-22.
